# Building Sustainable Rural Age-Friendly Communities: Grassroots Perspectives

**DOI:** 10.1093/geroni/igaa057.2448

**Published:** 2020-12-16

**Authors:** Patricia Oh

**Affiliations:** AARP, Bowdoinham, Maine, United States

## Abstract

Joining the AARP Network of Age-Friendly States and Communities does not make a community age-friendly; the age-friendly team must cultivate community engagement, develop collaborations with diverse stakeholders, mobilize resources, and document achievements. Little research describes the tools age-friendly rural communities use to effect change and develop sustainability. Thematic content analysis of 67 interviews conducted between December 09, 2018 and January 24, 2020 with age-friendly leaders in rural Maine communities suggested that peer-to-peer networking, privileging local knowledge, engaging local and regional partners, technical advice from a trusted source, and fun were among the tools used to move age-friendly rural work forward.

